# Quantitative optical measurement of mitochondrial superoxide dynamics in pulmonary artery endothelial cells

**DOI:** 10.1142/S1793545817500183

**Published:** 2018

**Authors:** Zahra Ghanian, Girija Ganesh Konduri, Said Halim Audi, Amadou K. S. Camara, Mahsa Ranji

**Affiliations:** * Department of Electrical Engineering, University of Wisconsin-Milwaukee, Milwaukee, Wisconsin, USA; † Department of Pediatrics, Division of Neonatology, Medical College of Wisconsin, Milwaukee, Wisconsin, USA; ‡ Department of Biomedical Engineering, Marquette University, Milwaukee, Wisconsin, USA; § Department of Anesthesiology and Anesthesia Research, Medical College of Wisconsin, Milwaukee, Wisconsin, USA

**Keywords:** Fluorescence microscopy, time-lapse imaging, oxidative stress, superoxide, pentachlorophenol sodium salt, rotenone, antimycin A, potassium cyanide, MitoSOX

## Abstract

Reactive oxygen species (ROS) play a vital role in cell signaling and redox regulation, but when present in excess, lead to numerous pathologies. Detailed quantitative characterization of mitochondrial superoxide anion ( $O_{2}^{•-}$) production in fetal pulmonary artery endothelia cells (PAECs) has never been reported. The aim of this study is to assess mitochondrial $O_{2}^{•-}$ production in cultured PAECs over time using a novel quantitative optical approach. The rate, the sources, and the dynamics of $O_{2}^{•-}$ production were assessed using targeted metabolic modulators of the mitochondrial electron transport chain (ETC) complexes, specifically an uncoupler and inhibitors of the various ETC complexes, and inhibitors of extra-mitochondrial sources of $O_{2}^{•-}$. After stabilization, the cells were loaded with nanomolar mitochondrial-targeted hydroethidine (Mito-HE, MitoSOX) online during the experiment without washout of the residual dye. Time-lapse fluorescence microscopy was used to monitor the dynamic changes in $O_{2}^{•-}$ fluorescence intensity over time in PAECs. The transient behaviors of the fluorescence time course showed exponential increases in the rate of $O_{2}^{•-}$ production in the presence of the ETC uncoupler or inhibitors. The most dramatic and the fastest increase in $O_{2}^{•-}$ production was observed when the cells were treated with the uncoupling agent, PCP. We also showed that only the complex IV inhibitor, KCN, attenuated the marked surge in $O_{2}^{•-}$ production induced by PCP. The results showed that mitochondrial respiratory complexes I, III and IV are sources of $O_{2}^{•-}$ production in PAECs, and a new observation that ROS production during uncoupling of mitochondrial respiration is mediated in part via complex IV. This novel method can be applied in other studies that examine ROS production under stress condition and during ROS-mediated injuries *in vitro*.

## 1. Introduction

Reactive oxygen species (ROS) are biologically important molecules. They are involved in signaling, but when present in excess (oxidative stress), they exert deleterious effects on cell structure and function. Cellular ROS are produced in mitochondria and from non-mitochondrial sources, including the NADPH oxidase (NOX) system and uncoupled endothelial nitric oxide synthase (eNOS). Mitochondrial ROS are generated primarily during electron transfer along the electron transport chain (ETC) complex proteins. Of all the cellular sources of ROS, electron leakage from the ETC to O_2_ (dioxygen) is responsible for a steady flux of superoxide ( $O_{2}^{•-}$) anions, which makes mitochondria the major site of the primordial ROS production. ^1–4^ Under physiological conditions, small amounts of ROS are generated due to partial reduction of O_2_ into $O_{2}^{•-}$ anion.^4–6^ The major areas for electron leak leading to $O_{2}^{•-}$ production includes flavins and quinones of the ETC complexes, and this is more prominent under conditions that decrease electron transfer to complex IV, the terminal electron acceptor.^4^

Physiological ROS are maintained at acceptable levels by endogenous matrix of antioxidant defense mechanisms. In this case, the $O_{2}^{•-}$ anions are readily dismutated to other radical species, such as hydrogen peroxide (H_2_O_2_) and sometimes to the more reactive hydroxyl radicals (OH^•^)^4^ by mitochondrial manganese superoxide dismutase (MnSOD) and other enzymatic reactions.^4,6^ Therefore, since $O_{2}^{•-}$ is the precursor of most of the downstream ROS and it is also involved in the propagation of oxidative stress-mediated damages, it has become an important biomarker to assess oxidative damage of key macromolecules.^6,7^

There is an expanding body of evidence that ROS participate in numerous cell signaling mechanisms, and are widely implicated in the pathologies of cellular and tissue injury.^8–15^ Understanding and discerning the role of ROS depends on the ability to measure and quantify the dynamics of mitochondrial ROS production under normal and stress conditions. A variety of metabolic modulators of mitochondria, such as ETC uncouplers and inhibitors, as well as metabolic stress conditions, for example, hyperoxia and hypoxia (O_2_ stress), lead to altered mitochondrial ROS production.^16–19^

MitoSOX Red, a fluorescence probe, and a derivative of Hydroethidine (HE), is a widely used probe for mitochondrial-targeted $O_{2}^{•-}$ detection in cultured cells.^14^ The positive charge on the phosphonium group in MitoSOX Red selectively targets this cell-permeant HE derivative to mitochondria.^20^ Once in mitochondria, MitoSOX is oxidized by $O_{2}^{•-}$ and the product elicits fluorescence response^20,21^ proportional to $O_{2}^{•-}$ concentration. In numerous studies, MitoSOX use was validated with fluorescent microscopy^21–30^ for selective detection of mitochondrial $O_{2}^{•-}$ in endothelial cells,^24,25,31^ cardiomyocytes, ^24,29^ fibroblasts,^30^ and neuronal cells.^21,27,28^ However, none of these studies used real-time monitoring of mitochondrial $O_{2}^{•-}$ production. Therefore, we aimed to develop a simple and quantitative method for simultaneous detection of mitochondrial $O_{2}^{•-}$ production in a large population of cultured cells using the mitochondrial $O_{2}^{•-}$ sensitive membrane permeable MitoSOX Red. To the best of our knowledge, we show for the first time that time-lapse microscopy in combination with nanomolar MitoSOX compartmentalized in mitochondria can be used to quantitatively measure mitochondrial $O_{2}^{•-}$ production in live healthy and diseased cells, in real-time. The other novelty of this study is localizing the primary source of $O_{2}^{•-}$ under stress conditions using the proposed dual agent protocol.

Since $O_{2}^{•-}$ is the primordial ROS, we devoted our effort in this study to monitor and quantify the dynamic changes in $O_{2}^{•-}$ production during modulation of ETC activities in PAEC, which to date has not been reported. Our method was based on the PAEC model and the use of modulators of the ETC; inhibitors. rotenone (complex I), antimycin A (complex III) and KCN (complex IV) and the ETC uncoupler, PCP. To further ascertain that $O_{2}^{•-}$ anions are primarily of mitochondrial source, the SOD mimetics, MitoTempol was used during the perturbation of mitochondrial ETC function. Addition of the metabolic agents induced metabolic stress that led to dynamic changes in the rate of $O_{2}^{•-}$ production over time. It is worth noting that in other cell types, especially nonexcitable cells like PAEC, other sources of ROS production, for example, NOX could contribute to the total $O_{2}^{•-}$ production during simulated metabolic/hypoxic stress. Therefore, additional experiments were conducted using apocynin to assess potential ROS production from NOX sources. Overall, the use of time-lapse microscopy provides an ideal approach to study the spatial and temporal changes in mitochondrial $O_{2}^{•-}$ production in real-time during metabolic stress in live cells. This method not only assesses the $O_{2}^{•-}$ production from the ETC, but also localizes the source of ROS in mitochondria and extra-mitochondrial sources. Lastly, the application of this approach is not limited to studying dynamic ROS production in real-time in PAECs, it can be used in other types of cells under normal and pathophysiological conditions.

## 2. Materials and Methods

### 2.1. Cell preparation

PAECs from normotensive fetal lambs (NFL) were isolated and characterized using techniques we described previously.^32,33^ Isolated PAECs were cultured in DMEM (Life Technologies) with 20% FBS (Life Technologies) and 1X antibiotic/antimycotic (Life Technologies) at 37°C in 21% O_2_, 5% CO_2_, balance N_2_. PAECs between passages 3 and 4 were used for our experiments, and were cultured (10^4^ cells/well) in 4-well chamber slides (Lab-Tek, VCAT) and kept in the incubator (21% O_2_, 5% CO_2_, 74% N_2_) at 37°C before imaging. At the onset of each experiment and fluorescent imaging, the cells were loaded with 200nM Hoechst (Life Technologies H1399, excitation/emission. UV/blue) in 2ml growth medium to stain the nuclei and then incubated for 30 min before imaging. Following incubation, the cells were washed twice, and Hank’s Balanced Salt Solution (HBSS, Life Technologies 14025092) was added to the dish for subsequent fluorescent imaging. During the imaging, mitochondrial $O_{2}^{•-}$ production was visualized in intact cultured PAECs loaded with a mitochondrial-targeted dihydroethidium (Mito-HE), red fluorescence probe (MitoSOX 500 nM, Invitrogen M36008; excitation/emission. 510/580 nm). Once in the mitochondria, MitoSOX is oxidized by $O_{2}^{•-}$ and exhibits a red fluorescent response proportional to the $O_{2}^{•-}$ level.

### 2.2. Live cell time-lapse imaging

#### 2.2.1. MitoSOX loading

Production of $O_{2}^{•-}$ in live cells was visualized by MitoSOX Red, a triphenylphosphonium (TPP^+^)-linked DHE compound. It is preferentially attracted to the mitochondria by > 100-fold compared with the cytosol^34^ due to the strong negative mitochondrial membrane potential (ΔΨm). In mitochondria, the accumulated MitoSOX is oxidized by $O_{2}^{•-}$ and the product exhibits fluorescence upon binding to mitochondrial DNA.^20,21^ The high matrix concentration of MitoSOX also allows the dye to compete with the endogenous mitochondrial $O_{2}^{•-}$ scavenger, MnSOD, for $O_{2}^{•-}$.^35,36^

For the time lapse monitoring of the $O_{2}^{•-}$ production, MitoSOX loading was performed online while the experiment was running in the microscope chamber environment. To maintain a high intra-mitochondrial concentration of MitoSOX, the loading process was not followed by washout of residual dyes. This approach is to ensure that the MitoSOX concentration (nM) in mitochondria is high enough to compete with MnSOD for $O_{2}^{•-}$. Ongoing binding to mitochondrial $O_{2}^{•-}$ allows for real-time monitoring of the MitoSOX oxidation rate, which reflects $O_{2}^{•-}$ concentration and rate of production over time.

#### 2.2.2. Fluorescent microscopy

Live cells were imaged using a Nikon Ti-E inverted microscope, with four fluorescent interchangeable filter cubes in addition to the standard DIC and bright-field channels. The bright field images were acquired using an overhead halogen lamp, whereas the fluorescent images used a mercury arc lamp, to take advantage of its intense peaks in the ultraviolet range. Each image was acquired at a magnification of 20 × with a scale of 0.32 *μ*m per pixel. The images were captured using a charge-coupled device camera (Q-imaging, Aqua Exi, 14 bit, 6.45 *μ*m per pixel) with exposure time (0.68 *μ*s/pixel in red channel) set to ensure proper use of the dynamic range of the camera, while avoiding saturation and photo bleaching. The filter set in the blue and red channels filters excitation spectra at 340–380nm and 528–553 nm, respectively with emission spectra at 435–485nm and 590–650 nm, respectively.^2^

Time-lapse images were obtained in the blue (Hoechst), red (MitoSOX), and bright field (BF) channels to monitor nuclei, mitochondrial ROS levels and the structure of the cells, respectively. Four fields of view (FOV) of cells were imaged (one FOV in each chamber of the bottom-glass dish) under the aforementioned settings. The microscope is surrounded by a custom-made chamber (Okolab) housed around the stage, providing gas exchange and controlled temperature for time-lapse imaging over several hours. During the experiments, the level of the CO_2_ inside the chamber was maintained at 5% by mixing CO_2_ with room air at the proper ratio and chamber temperature was kept at 37 ± 1 (SE)°C. O_2_ and CO_2_ levels were continuously monitored with an O2-BTA model O_2_ sensor and CO_2_-BTA model CO_2_ probe (Vernier Co., Beaverton, OR).

#### 2.2.3. Co-localization

Z-stacks of green and red images of 20 randomly selected PAECs previously stained with Mitotracker green (50 nM) and loaded online with MitoSOX red (500 nM) were acquired. The nuclear region of each cell image in *z*-stacks was excluded using the nuclear mask obtained in blue channel. Co-localization analysis was performed by pseudocoloring and merging green and red fluorescence images together. Both fluorophores resided within the same 3D volume whose minimum size is defined by the resolution limits of the microscope (0.32 *μ*m at a magnification of 20 ×). Quantitative statistical analyses of both the spatial distribution and the correlation between the intensities of the green and red fluorescence images were performed to measure co-localization. Co-localization was determined by quantification of overlapping channels, performed by using the “object-based methods” algorithm of JACoP plugin V2.0.^37^

#### 2.2.4. Experimental protocols

Our experimental protocol was designed to measure changes in mitochondrial $O_{2}^{•-}$ production associated with metabolic stress conditions as a model of ROS production in PAECs. Time-lapse images of FOV were captured in blue, red, and bright field channels, all in 1-min intervals for 80 min. Ten minutes of baseline imaging was followed by the addition of MitoSOX (500 nM) to the PAECs. Imaging was continued for 20 min after the addition of MitoSOX. Then the cells were treated with metabolic stressors, including pentachlorophenol sodium salt (PCP, ETC uncoupler, 15 *μ*M, Sigma Alrich #76480), rotenone (Complex I inhibitor, 15 *μ*M, Sigma Alrich #R8875), antimycin A (Complex III inhibitor, 1 *μ*M, Sigma Aldrich #A8674), or potassium cyanide (KCN, complex IV inhibitor, 20 *μ*M, Sigma Aldrich #60178) to study the $O_{2}^{•-}$ production in the mitochondria as indicated by fluorescence intensity. The aforementioned concentrations of these mitochondrial modulators were chosen based on dose response studies, with the goal of achieving sufficient inhibition or uncoupling (data not shown) while minimizing their toxic effect as shown in the cell viability studies. Image acquisition continued for 50 min after adding the stressors. These agents also provide means to validate mitochondrial $O_{2}^{•-}$ production in PAECs during online assessment of MitoSOX oxidation based on our experimental protocol.

To demonstrate the feasibility of our method to localize the source of mitochondrial $O_{2}^{•-}$ under the metabolic stress conditions, dual agents (a combination of metabolic modulators) experimental protocols were designed. For instance, 5 min after initiation of uncoupling induced by the addition of PCP, a metabolic inhibitor (rotenone (ROT), antimycin A (AA) or KCN) was administered. This approach was to identify the specific ETC complexes associated with the large increase in mitochondrial $O_{2}^{•-}$ production during uncoupling (PCP) of mitochondrial respiration. Fluorescent recording was for 50 min after adding the second metabolic agent/modulator. The dual agent experimental protocol was also helpful to validate mitochondrial $O_{2}^{•-}$ production with real-time monitoring of MitoSOX oxidation using positive controls (uncoupler or inhibitor) as first agent and negative control (MitoTempol) as second agent.

Viability of PAECs treated with MitoSOX only, MitoSOX and PCP, MitoSOX and PCP and KCN was assessed by trypan blue exclusion assay.^38^ Furthermore, additional experiments revealed that none of the mitochondrial modulators at the used concentrations interfered with MitoSOX fluorescent emission signal.

### 2.3. Image and signal processing

#### 2.3.1. Cell segmentation

Our previously reported cell segmentation algorithm^39^ was used to detect the border of the cells in the bright field images. The obtained mask (cells contours) was applied to the time-lapse image stack in the red fluorescent channel. The nuclei were also identified in the blue images and the resulting blue binary image was used as a mask for the stack of the red images to exclude the nuclei contribution to the red intensity profiles. The mean intensity of the mitochondria in red channel images was calculated as a raw intensity profile of the live PAECs over time. This profile helps to monitor the dynamics of the mitochondrial ROS production before, during and after altering ETC function.

##### Intensity profile

**Fig. 1:**
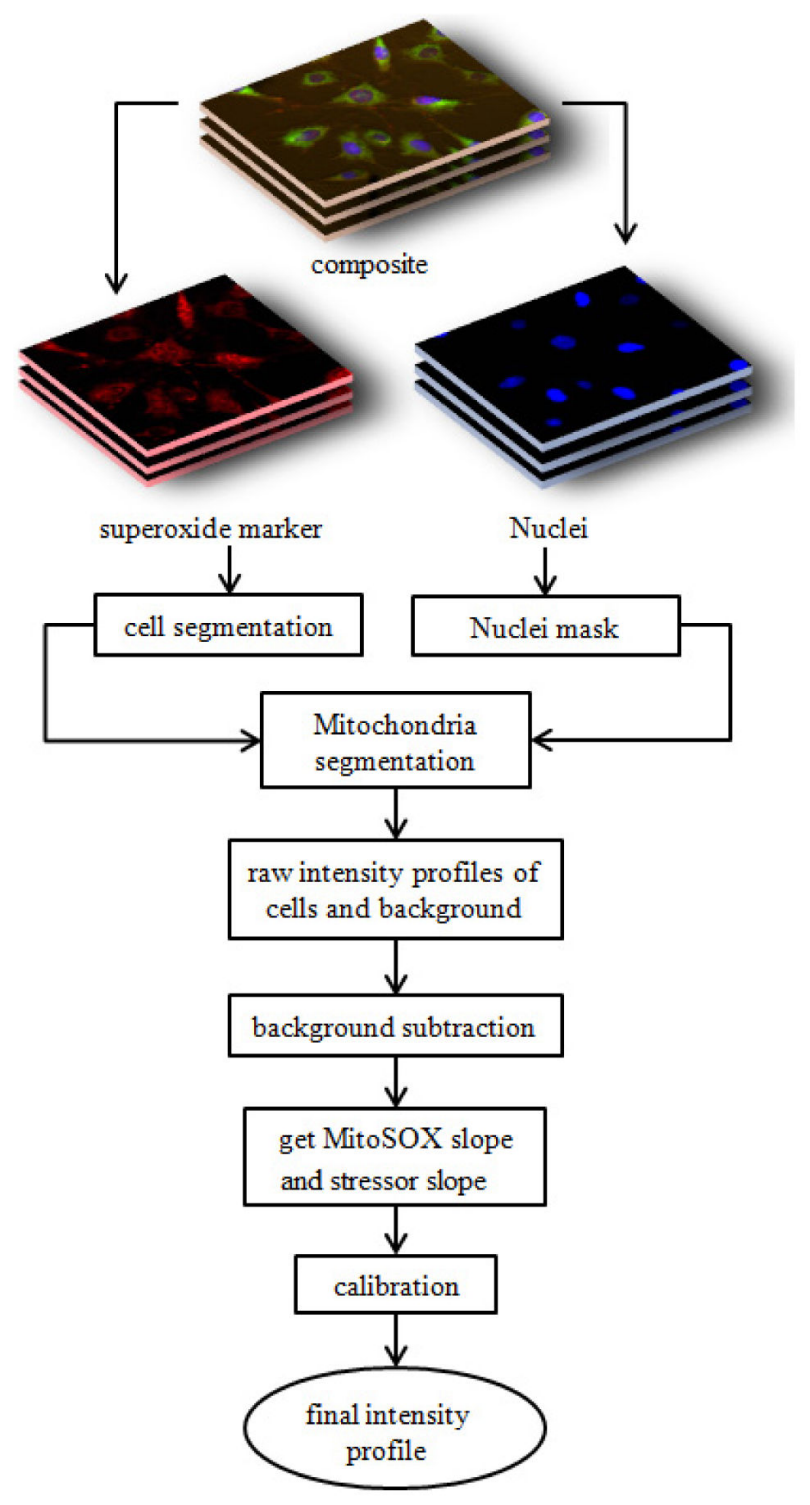
A schematic illustration of the methodology used to obtain the MitoSOX fluorescent intensity profiles of the cells from the input fluorescent images. The schematic flow chart shows the same approach for a typical single or dual agent experiment.

Figure 1 represents the overall methodology to obtain final intensity profiles of the cells from the input fluorescent images. Intensity profile of the red fluorescence images shows the dynamic of $O_{2}^{•-}$ production in response to MitoSOX (*t* = 10 min) and metabolic agents (*t* = 30 min). In order to quantify the dynamic changes in mitochondrial $O_{2}^{•-}$ levels and compare the changes in $O_{2}^{•-}$ production between the control group (PAECs with MitoSOX, i.e., no treatment) and treated groups (PAECs treated with inhibitors or/and the uncoupler), the raw intensity profiles were first background subtracted and then slope calibrated. Day-to-day variation of light intensity and illumination pattern led to variations in the basal level intensity, which was accounted for background subtraction. A linear scaling, slope calibration method, which preserve the slope ratio (SR), was employed for better demonstration and comparison. The slope calibration was done in the single agent/modulator experiments, using the linear property of the MitoSOX-induced intensity rate (for 20 min interval after administration of MitoSOX and before addition of the metabolic agent). The slope of the linear fit of the intensity profiles was calculated in the MitoSOX interval (*t* = 11–29 min) for both control and treated groups. The MitoSOX-interval slope of the cell intensity profile in both control (no treatment) and treated groups were calibrated based on the MitoSOX-interval slope of a control in such a way that their slopes at this interval and before adding a metabolic agent were the same; the difference in the slopes of the control and treated groups were distinguishable after adding the agent. Therefore, the resulting intensity profiles are visually comparable for control and treated groups in the time interval of *t* = 30–80 min, which is helpful to evaluate the effect of the metabolic agent on the intensity profile. As mentioned earlier, for the dual agent experiments, the second agent, rotenone, antimycin A or KCN was administrated 5 mins after addition of PCP. Since the objective of the dual agent experiments is the evaluation of the inhibitor effect in the presence of the PCP, the slope calibration was applied based on the PCP-interval slope (*t* = 33–35 min) in such a way that their slopes at this interval and therefore before adding the second agent were the same. The resulting intensity profiles of the dual agent groups were compared to the fluorescent intensity profiles of the PCP only group in the time interval of *t* = 35–80 min, to evaluate the effects of ETC inhibitors (ROT, AA and KCN) on the rate of fluorescent change after maximally oxidizing the ETC with PCP.

### 2.4. Quantification of the kinetics of superoxide production

Exponential and sigmoidal empirical functions were used for quantitative description of the red fluorescence time-intensity curves measured in treated and control cells, respectively.

(1) $$I_{1}(t)\sim a+b*e^{-\frac{t-30}{T}},$$

(2) $$I_{2}(t)\sim \frac{c}{1+d*e^{-\frac{t-30}{\tau }}}.$$

For the exponential function (Eq. (1)), the parameters are amplitude (*b* in F.A.U.), time constant (*T* in min), and the constant intensity offset (*a* in F.A.U.). For the sigmoidal function (Eq. (2)), the parameters are amplitude (*c* in F.A.U.), time constant (*τ* in min), and the displacement factor *d*. These parameters were estimated by fitting (MATLAB, MathWorks, Inc. Massachusetts 01760 USA) Eqs. (1) or (2) to the red fluorescence time-intensity profiles from treated, and control groups, respectively.

The estimated values of the above parameters can be used to quantify the initial surge in $O_{2}^{•-}$ production in the presence and absence of metabolic agents/modulators. For instance, the parameter groups $\frac{-b}{T}$ and $\frac{\mathrm{cd}}{\tau {\left(1+d\right)}^{2}}$ are measures of the initial rates (F.A.U. per min) of $O_{2}^{•-}$ production in the presence and absence of the metabolic agents, respectively.

### 2.5. Statistical analysis

Data are shown as means ± SE. Student’s *t*-test was used for normally distributed data. A *p* value < 0.05 was considered significant.

## 3. Results

Co-localization of MitoTracker green signal with the MitoSOX red signal in PAECs confirmed that $O_{2}^{•-}$ anions were produced from mitochondria in cells exposed to metabolic stress conditions. The degree of co-localization of these two fluorescent signals in the PAECs was 0.91 ± 0.06, indicating significant mitochondrial localization of the $O_{2}^{•-}$ production.

Viability of PEACs treated with MitoSOX only, MitoSOX plus PCP, and MitoSOX plus PCP and KCN was assessed at the end of the 80 min experimental protocol. The percentages of cells that were viable following the aforementioned treatments were. 88 ± 2%, 76 ± 3%, and 80 ± 3%, respectively.

**Fig. 2:**
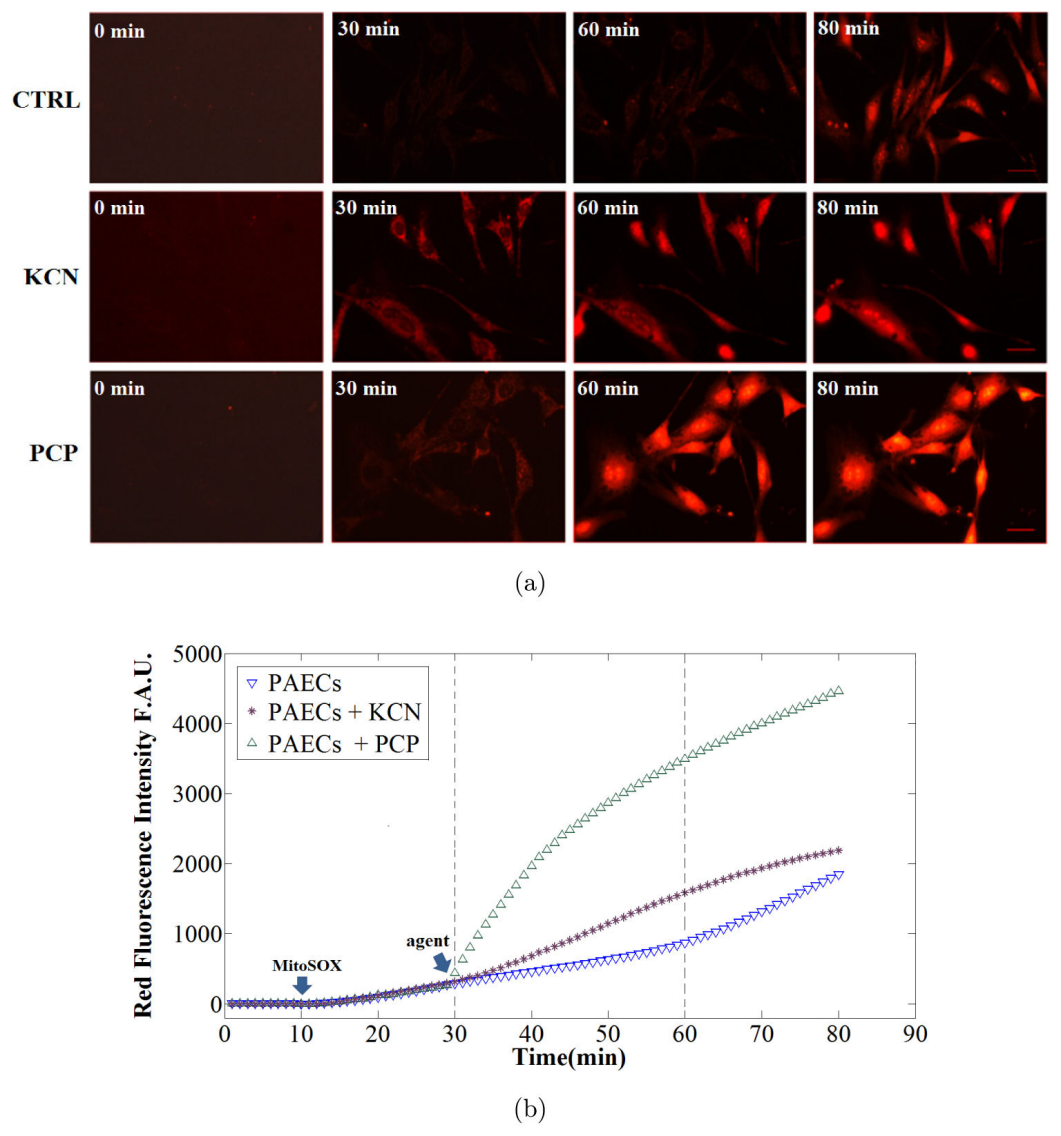
Panel (a) representative raw frames from image stack of time-lapse microscopy showing dynamic $O_{2}^{•-}$ production in three experiments using live PAEC loaded with MitoSOX in control (no treatment), in the presence of complex IV inhibitor (KCN) or the uncoupler PCP. Elapsed time is indicated in the upper left corner of each frame (0, 30, 60 and 80 min). Note that the scale bar in the right corner of the right bottom frame is the same for all frames and is 32 *μ*m. Panel (b) representative dynamic recordings of mean fluorescence intensity profiles of the cells in the three aforementioned conditions. The blue curve displays the dynamic of the fluorescence intensity in control cells over time; the green and purple curves represent the fluorescence intensity in cells treated with PCP and KCN, respectively. The arrows indicate the time MitoSOX and agents were added to the cells. F.A.U., fluorescence arbitrary unit; KCN, potassium cyanide; PCP, pentachlorophenol sodium salt.

The top panel in Fig. 2(a) displays red fluorescent images of PAECs, from which, intensities were translated to the rate of $O_{2}^{•-}$ production in mitochondria. The first row is the control (CTRL) group, the second and third rows are cells with the addition of an uncoupler (PCP), and an inhibitor (KCN), respectively. The first column of images is the first frame of the time-lapse imaging (*t* = 0 min) showing the FOV of interest with no contrast between cells and background. The second column of images shows the same FOV just before adding the uncoupler or inhibitor at time *t* = 30 min. The addition of the agents enhances the contrast between cells and background due to increasing $O_{2}^{•-}$ production in mitochondria, as evidenced by the increased MitoSOX (added at *t* = 10 min) fluorescent signal. Compared to the second column, the third column images show the frames (at time *t* = 60 min) with slight increase in the intensity in the first row (CTRL group), and significant increase in intensity in the second and third rows due to exponential increase in $O_{2}^{•-}$ production after adding KCN and PCP, respectively. The last column of images shows the frames at *t* = 80 min demonstrating larger increase in $O_{2}^{•-}$ production rate in the control group (first row), and smaller increase in $O_{2}^{•-}$ production in the treated groups (second and third rows). These observations are consistent with the fluorescent intensity profiles demonstrated in panel (b). Figure 2(b) shows the corresponding quantitative changes in the fluorescence intensity in the cell after calibration. Inhibiting and uncoupling mitochondria (purple and green curves, respectively) significantly increased the rate of $O_{2}^{•-}$ production when compared to the control (CTRL) rate of $O_{2}^{•-}$ production. Figure 2(b) also demonstrates that uncoupling the mitochondrial ETC with PCP resulted in an abrupt and marked increase in the rate of $O_{2}^{•-}$ production when compared to the KCN treated cells. Enhanced fluorescence intensity was evident right after addition of the metabolic agents, and the intensity increased continuously in an exponential manner over time, demonstrating a time-dependent amplification of ROS production.

**Fig. 3:**
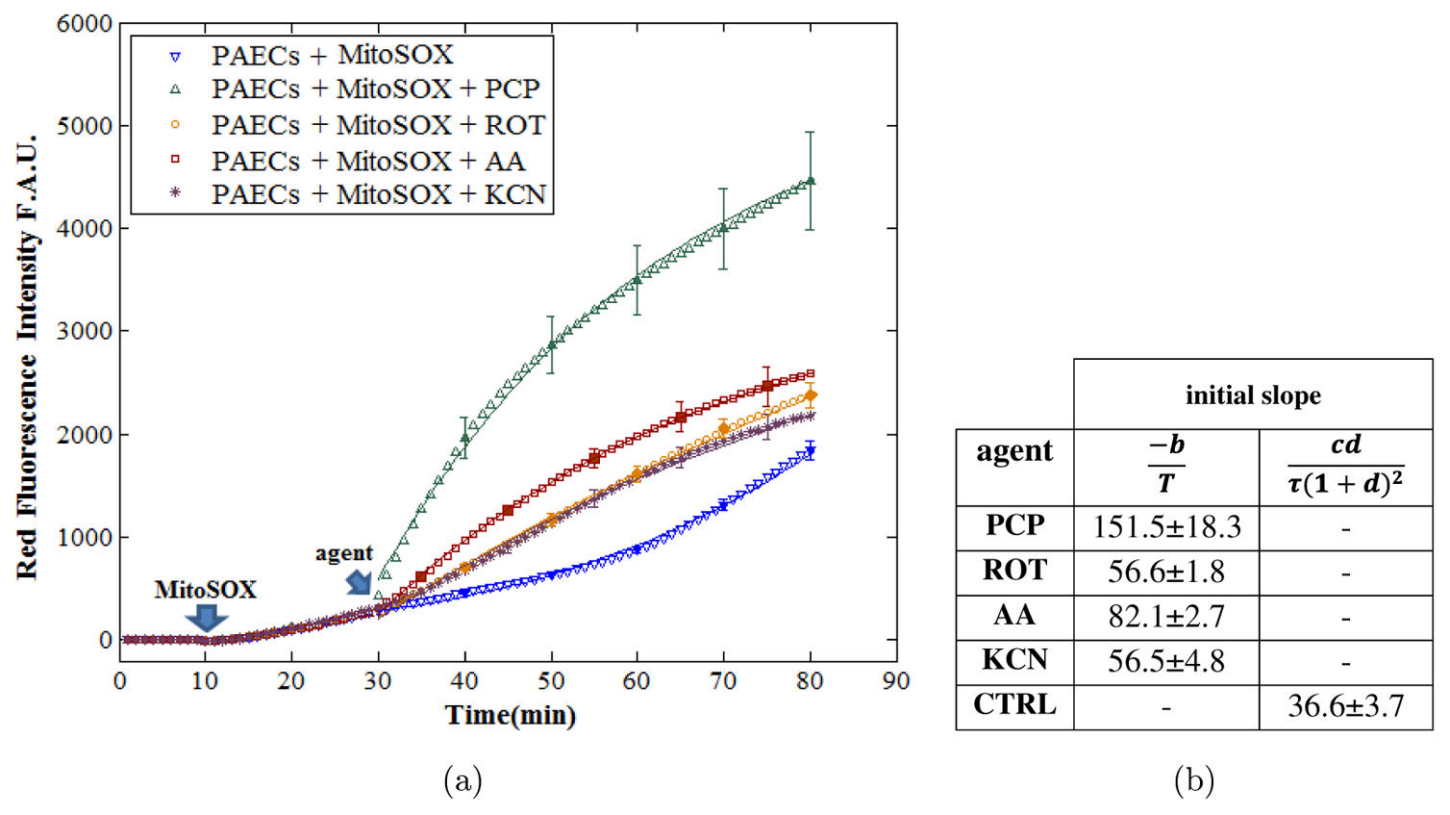
(a) Dynamic fluorescence intensity profiles over time of cells in the presence or absence of the uncoupler (PCP) or the mitochondrial ETC complex inhibitors (ROT, AA, or KCN). Values are means ± SE; *n* = 6 for each treatment group. The solid lines are exponential and sigmoidal fits to the mean values of the agent-treated and control data. (b) The table shows the mean values of initial slope (initial rates of superoxide production) of the exponential and sigmoidal fits at *t* = 30 min for each treatment group and control. F.A.U., fluorescence arbitrary unit; ROT, rotenone; AA, antimycin A; KCN, potassium cyanide; PCP, pentachlorophenol sodium salt.

**Fig. 4:**
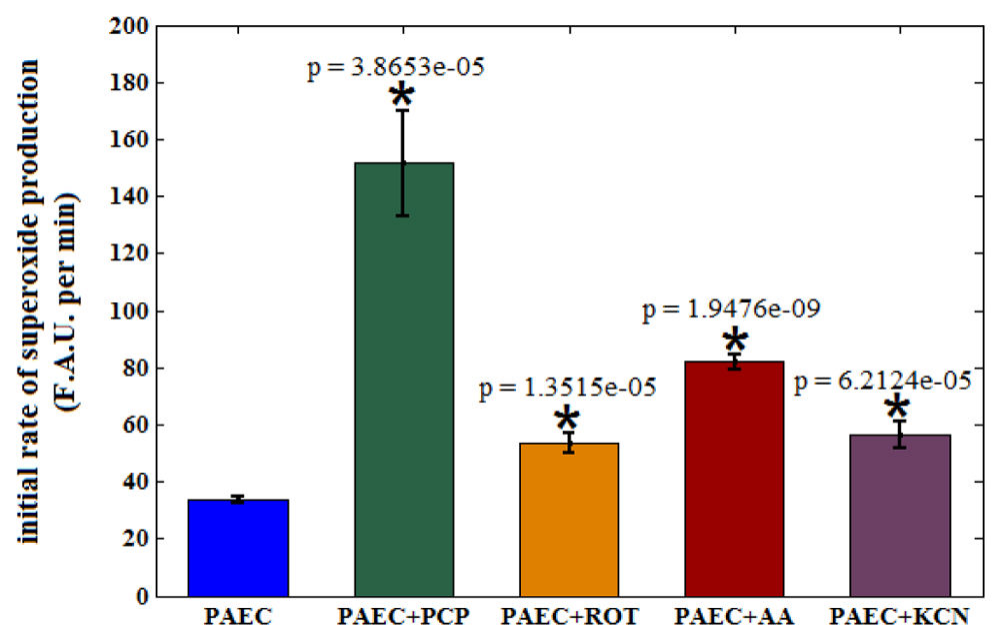
Summary bar graphs show the slope of the fluorescence intensity profiles right after the agent administration. Bar graphs show the means ± SE of the red fluorescence intensity slopes (*t* = 30 min) for the five groups of cells. Addition of agents to PAECs (green, orange, red, and purple bars) resulted in significant (^*^p < 0.05) increase in the rate of $O_{2}^{•-}$ production when compared to the nontreated (control) PAECs (blue bar). *n* = 6/group.

To quantify the rate of $O_{2}^{•-}$ production in the absence and presence of inhibitors of the ETC or the uncoupler, PCP, (*t* = 30–80 min), sigmoidal and exponential empirical equations were fitted to the experimental data (control and treated cells). Figure 3 shows the initial slope of the intensity profile right after addition of the uncoupler/inhibitor. These nonlinear fits demonstrate that treating the cells with an oxidizing agent (PCP 15 *μ*M), or reducing agents (ROT 15 *μ*M, AA 1 *μ*M, and KCN 20 *μ*M) results in significantly larger increase in the initial rate of $O_{2}^{•-}$ production (151.5 ± 18.3, 56.6 ± 1.8, 82.1 ± 2.7, 56.5 ± 4.8) at *t* = 30 min when compared to that of the control cells (36.6 ± 3.7). Presented values correspond to the initial time (*t* = 30 min) when the metabolic agents were added. To evaluate for consistency and reproducibility of the results, a total of 48 wells of PAECs were imaged, with *n* = 6 for each of the treated groups and the control. The slopes of the fitted functions were calculated at the time of the administration of the agent for six FOVs per group for the treated and control cells. The green curve of Fig. 3 corresponds to the average profile of the PCP experiments, and it demonstrates the fastest and greatest increase in the rate of $O_{2}^{•-}$ production (4.90 ± 0.5) in uncoupled mitochondria. The slopes of the intensity right after the addition of mitochondrial metabolic modulators (*t* = 30 min) were compared statistically in Fig. 4, demonstrating significant changes between treated groups versus the control group. Increase in the slope intensity profile by a factor of 1.83 ± 0.05 (orange curve, Fig. 3 and orange bar, Fig. 4) reflects the ROT-induced increase in the rate of $O_{2}^{•-}$ production from complex I. The red and purple bars in Fig. 4 show greater rates of $O_{2}^{•-}$ production by AA and KCN, respectively, by a factor of 2.66 ± 0.08 and 1.83 ± 0.14 compared to the control group.

**Fig. 5:**
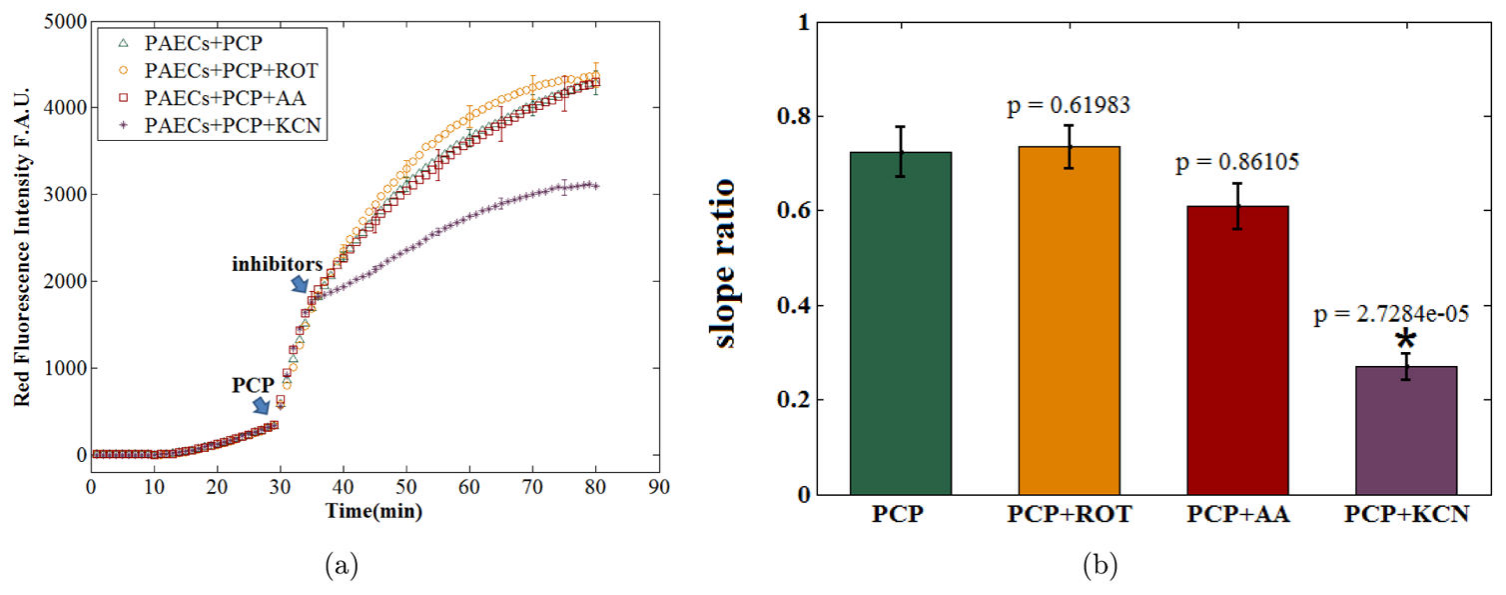
(a) Mean ± SE of fluorescence intensity profiles of $O_{2}^{•-}$ production over time in PAECs treated with PCP alone or PCP with one of the ETC inhibitors; (b) Summary bar graphs show mean ± SE of the slope ratio of the red fluorescence intensities for the four treatment groups *n* = 6/group.

To confirm our method for localizing the mitochondrion as the source of $O_{2}^{•-}$ production under metabolic stress conditions, a dual agent exposure protocol was designed. This method was devised to partition the $O_{2}^{•-}$ production from different parts of the ETC. After oxidizing the ETC with PCP, the ETC was reduced with the different ETC complex inhibitors (complexes I, III and IV) to tease out the complex/s responsible for the surge in $O_{2}^{•-}$ production when PCP fully oxidized the ETC. The curves in Fig. 5 represent the profiles of mean intensity in each time point for the corresponding groups. The green curve displays PCP-induced intensity profile compared to the orange, red, and purple curves, which demonstrate the effects of the addition of ROT, AA, and KCN to the PCP treated cells, respectively. The SR was calculated for all four groups, with *n* = 6 per group. Bar graphs in Fig. 5 show the means and SEM of the SR of the red fluorescence intensity for each of the four groups of cells. When compared to the addition of ROT and AA (orange and red bars, respectively), the addition of KCN to PCP-treated PAECs (purple bar) resulted in a significant reduction (*p* = 9.8026e-5), 62.7%, in the rate of the fluorescence intensity as compared to the fluorescence intensity of PAECs treated with PCP alone (green bar). This result shows that only KCN was able to significantly attenuate the $O_{2}^{•-}$ production by the uncoupling agent PCP.

**Fig. 6:**
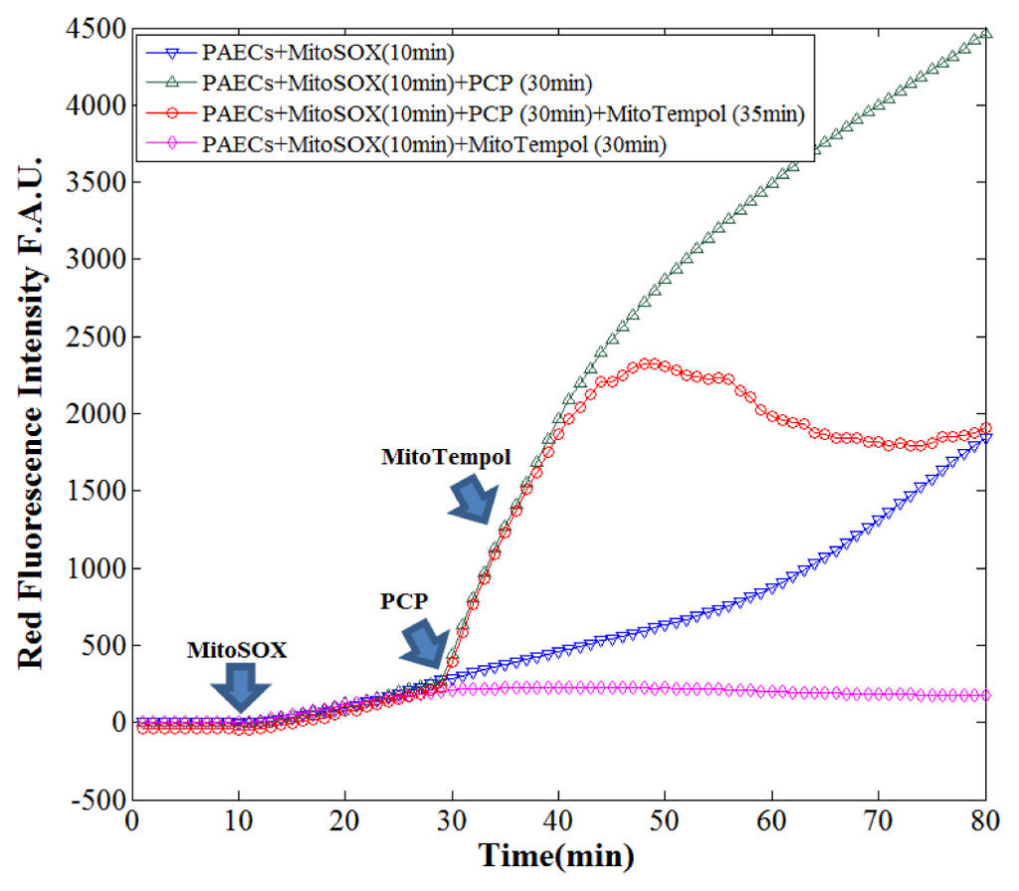
Representative fluorescence intensity profiles in untreated cells (control) and PCP treated cells in the absence and presence of the superoxide anion scavenger MitoTempol over time. The arrows indicate the time MitoSOX (10 min), PCP (30 min), and MitoTempol (35 min) were added to the cells. F.A.U., fluorescence arbitrary unit; this panel shows that MitoTempol reversed PCP-induced $O_{2}^{•-}$ production.

To provide further evidence that the surge in $O_{2}^{•-}$ production by PCP was mainly from mitochondria, the mitochondrial-targeted ROS scavenger, MitoTempol was used. The dual agent exposure protocol was used to validate mitochondria as the primary source of $O_{2}^{•-}$ production during real-time $O_{2}^{•-}$ oxidation of MitoSOX, using the uncoupler PCP followed by MitoTempol. Figure 6 shows fluorescence intensity profiles in untreated cells (control; blue curve) and PCP treated cells in the absence and presence of MitoTempol over time (green and red curves, respectively). As observed previously, adding PCP at *t* = 30 min markedly increased $O_{2}^{•-}$ production rate (compare green profile with blue curve); adding MitoTempol at *t* = 35 min significantly decreased the rate of $O_{2}^{•-}$ production (red profile versus green curve), and by *t* = 75 min, the PCP-induced $O_{2}^{•-}$ production was completely abolished, reaching $O_{2}^{•-}$ levels similar to the control cells (blue curve). The pink curve in this panel shows that using MitoTempol decreased the rate of $O_{2}^{•-}$ production when compared to the control cells (blue curve). Comparison of the pink and blue curves confirms that fluorescence measurement of MitoSOX oxidation reflects the dynamic changes in mitochondrial $O_{2}^{•-}$ production in real-time. This result further signifies that mitochondria are the primary source for $O_{2}^{•-}$ production in the PAECs during metabolic stress. In addition, we also observed that apocynin, a NOX inhibitor, did not alter the trajectory of the mitochondrial $O_{2}^{•-}$ production during metabolic stress (data not shown), further confirming that the primary/major source of ROS during metabolic modulation in the PAECs is mitochondria.

## 4. Discussion

This study aimed to quantify mitochondrial $O_{2}^{•-}$ production dynamics in cultured endothelial cells undergoing metabolic stress, utilizing fluorescent time-lapse microscopy. Experimental protocols were designed to (1) localize the source of the mitochondrial $O_{2}^{•-}$, (2) measure the changes in mitochondrial $O_{2}^{•-}$ levels and rate of production, (3) quantify transient behavior of $O_{2}^{•-}$ production during disruption of the ETC to mimic pathological ROS emission (production > scavenging). A major finding of this study is that ETC inhibitors and uncouplers, when given separately, induced exponential increase in the level $O_{2}^{•-}$ production. Complex IV seems to play a major role in ROS production during uncoupled mitochondrial respiration.

The mitochondria-specific indicator MitoSOX Red was used to monitor $O_{2}^{•-}$ production. This probe has been validated by others^21,24,25,27,29,40,41^ and for selective detection of mitochondrial $O_{2}^{•-}$ production in endothelial cells.^24,25,31^ However, online loading of ECs with nanomolar MitoSOX and real-time monitoring of mitochondrial $O_{2}^{•-}$ production have not been previously reported.

Although several recent studies have been conducted with live cell imaging^42^ and with plate reader assays,^43^ none of these studies provided real-time monitoring of mitochondrial $O_{2}^{•-}$ dynamic production over time. The novelty of our method is the real-time monitoring of the $O_{2}^{•-}$ dynamics in mitochondria, during targeted alteration of ETC function over time. Our current study mainly focused on mitochondrial sources of $O_{2}^{•-}$, by compartmentalizing the fluorescent probe in mitochondria, while the other methods, including HPLC,^44^ provide only global cellular information at fixed time-points. The other novelty of our study is localizing the primary source of the $O_{2}^{•-}$ in the uncoupled ETC. It is worth noting that most plate reader-based assays have poor sensitivity and are subject to artifacts.^45,46^ Use of plate reader systems for real-time kinetic measurements is associated with many pitfalls and limitations, including erroneous sustained increases in fluorescence, limited sensitivity and lack of selectivity, and single cell resolution. The HPLC method also has some limitations, including low throughput, and inability to track real-time changes in intracellular and extracellular $O_{2}^{•-}$ levels.

The measurement of various ROS is dependent on suitable techniques, which is currently hampered by the lack of sensitive and specific assays.^4^ Sub-micromolar MitoSOX is a sensitive and specific fluorescent marker for mitochondrial $O_{2}^{•-}$ and useful in monitoring the dynamic changes in $O_{2}^{•-}$ production during stress. MitoSOX, a cationic probe, selectively targets and enters mitochondria because of their strong negative ΔΨm.^4^ This biophysical attribute makes sub-micromolar/nanomolar Mito-SOX a suitable dye to assess mitochondrial-derived $O_{2}^{•-}$ production in live cells,^20^ including PAECs.

The ETC drives electrons from reduced coenzymes (NADH(H^+^) and FADH_2_) to O_2_, which undergoes the complete reduction to H_2_O catalyzed by complex IV. During the electron transfer, some electrons escape from the ETC at discrete sites to generate $O_{2}^{•-}$.^4^ $O_{2}^{•-}$ production is favored in general by high ΔΨm and large NADH(H^+^), or when electron transfer is impeded by alteration in the ETC complexes.^6,47^ In this scenario, a decrease in ROS production would portend ΔΨm depolarization due to enhanced electron transfer, as observed with uncoupling agents. Paradoxically, though, conditions have been reported in which mitochondrial uncoupling and ΔΨm dissipation are associated with increased production of ROS.^48^ This observation is consistent with our results which show PCP, an uncoupling agent, increases $O_{2}^{•-}$ production (Figs. 2–4). According to the proposed model of redox-optimized ROS balance by Aon *et al.*,^49^ this apparent paradox might be explained by the hypothesis that physiological signaling ROS occurs within an optimized redox state, and oxidative stress can happen at the extreme of either reduction or oxidation of the ETC. Consistent with this hypothesis, our results demonstrated higher $O_{2}^{•-}$ levels and rates of production in both the reduced (inhibited) and oxidized (uncoupled) ETC. A plausible explanation is that the elevated levels of $O_{2}^{•-}$ initially overwhelm the scavenging potential of mitochondrial antioxidant system and leads to excess ROS emission (oxidative stress).

Complexes I and III are fully reduced when they are blocked with ROT and AA, respectively. The highly reduced redox state creates a buildup of electrons along the ETC, leading to forced electron leak that reduces O_2_ to $O_{2}^{•-}$ anions; thus making complexes I and III as major sites of $O_{2}^{•-}$ production in the presence of ROT^50–52^ and AA.^20,21,53–58^ This observation is consistent with our results that show (Figs. 3 and 4) an increase in the intensity of red fluorescence emitted from mitochondria after the addition of rotenone or AA. KCN is an inhibitor of the enzyme cytochrome *c* oxidase or complex IV.^59,60^ The binding of cyanide to cytochrome *c* prevents the transfer of electrons from the enzyme to O_2_, creating a buildup of electrons in the ETC. As a result, inhibiting complex IV increases ROS production^4,61–63^ and decreases O_2_ availability for cellular respiration (hypoxia). Our data show that inhibition of O_2_ binding with complex IV leads to both electron backup and availability of O_2_ to be reduced to $O_{2}^{•-}$, without the generation of H_2_O. Moreover, inhibition of mitochondrial ETC by cyanide possibly increases $O_{2}^{•-}$ production at complexes I and III.^64–66^

Pentachlorophenol (PCP) is a powerful uncoupler of oxidative phosphorylation and also induces oxidative stress to cause mitochondrial damage. ^67–70^ PCP dissipates the proton gradient by consuming the proton motive force via increased proton leak back into the matrix. A recent study reported that PCP oxidizes the ETC and increase the activity of complex I,^71^ leading to the production of more protons and transfer of more electrons along the ETC. Lack of proton gradient for phosphorylation and polarized ΔΨm, activates a mechanism to compensate for the uncoupling effect of PCP, by increasing proton pumping and respiration, in an attempt to reestablish the proton gradient. Ironically, the increase in activity of the complexes in the uncoupled chain, increases electron transfer along the ETC, and as a result, increases electron leak to O_2_ leading to $O_{2}^{•-}$ production. ^49,72–75^ This notion is supported by our observation that uncoupling respiration with PCP led to the fastest and greatest increase (Fig. 3) in the mitochondrial $O_{2}^{•-}$ production. The finding also suggests that the increased protonophoric effect of the uncoupler (electron leak) leads to a feed-forward mechanism that exacerbates ROS production.

Administration of the metabolic stressor/modulators (PCP, KCN, AA, or ROT) in PAECs induces two phases of $O_{2}^{•-}$ production (Figs. 2 and 3). The initial phase is marked by immediate increase in $O_{2}^{•-}$ production following the addition of metabolic agents (surge phase); this is followed by smaller rate of $O_{2}^{•-}$ production (decrease in slope intensity) and eventually reaches the steady-state phase. This steady-state phase is consistent with the view that increased $O_{2}^{•-}$ level may induce MnSOD activity as a negative feedback mechanism to dismutate the excess $O_{2}^{•-}$ and therefore decrease the rate of $O_{2}^{•-}$ production over time^76^ as compared to the early phase of the stress condition. The fast dismutation of $O_{2}^{•-}$ by MnSOD also enhances the time course to attain the steady-state. It is worth noting that the oxidation of MitoSOX is 500 times slower than the rate of $O_{2}^{•-}$ scavenging by SOD.^21^ Therefore, less $O_{2}^{•-}$ is available for oxidizing Mito-SOX, which leads to a decrease or no change in the rate of the $O_{2}^{•-}$ production at the later stage of the imposed metabolic stress conditions (Figs. 2(b) and 3(a)).

Figure 3 also exemplifies the fits of the nonlinear empirical equations to the intensity profiles of the treated and non-treated PAECs. These nonlinear fits show that ETC uncoupling and inhibiting involves an exponential up-regulation of $O_{2}^{•-}$ production, while the nontreated cells show sigmoidal profile. Having these nonlinear fits of the intensity profiles allows for quantifying important parameters of $O_{2}^{•-}$ dynamics including $O_{2}^{•-}$ production rate, which is the slope of the fit in each time point and the $O_{2}^{•-}$ level that is proportional to the intensity value of the fit at each time point. Moreover, nonlinear fits provide the time constant (T; the time point when the intensity level rises to 65% of the ultimate value, b) of the exponential increase in the mitochondrial $O_{2}^{•-}$ levels. We also used the slope of the fit exactly after addition of the agent to quantify the initial rate of $O_{2}^{•-}$ production after each treatment. The nonlinear fits suggest that uncoupling of the mitochondrial ETC by PCP leads to the fastest rate of $O_{2}^{•-}$ production (bottom panel of Fig. 3) and highest levels of $O_{2}^{•-}$ accumulation compared to the inhibition of the ETC. A significantly higher slope of the exponential fit for the PCP-treated cells indicates faster $O_{2}^{•-}$ generation in the uncoupled ETC. Moreover, the exponential fit shows higher levels of accumulated $O_{2}^{•-}$ levels in PAECs with uncoupled ETC, which is consistent with the studies of Aon *et al.*^49^

The rate of $O_{2}^{•-}$ productions was also assessed in dual agent (combination of metabolic modulators) protocols to identify the complexes that mediate the uncoupling effect of PCP on $O_{2}^{•-}$ production, and the scavenging potential of mitochondrial-targeted scavenger (MitoTempol). $O_{2}^{•-}$ productions from mitochondria are primarily from complexes (I, III, and IV), distinguished by the use of specific blockers (see Materials and Methods) of the ETC complexes. Of the three complexes targeted, only complex IV blocker, KCN, significantly decreased the rate of $O_{2}^{•-}$ production induced by PCP. This suggests strongly that the uncoupling effect of PCP on $O_{2}^{•-}$ production is related to increased electron leak at the final acceptor complex IV to O_2_, without generation of H_2_O. Thus, in the uncoupled mitochondria, the main source of $O_{2}^{•-}$ production is probably associated with the oxidation of mitochondrial ETC via complex IV. Therefore, a notable observation is that in the PAECs, complex IV is a major site for $O_{2}^{•-}$ production when mitochondrial respiration is uncoupled.

Consistent with the redox-optimized ROS balance hypothesis, our method has the potential to model redox as ROS modulator and confirm the important role that redox modulation plays in controlling ROS production and potentially, ROS-mediated mitochondrial dysfunction and concomitant cellular injury. The single agent experiments showed that reduction (inhibition) or oxidation (uncoupling) of ETC leads to exponential increase in $O_{2}^{•-}$ production, with more pronounced effect in the presence of PCP (Fig. 3(a)). Therefore, any shift toward oxidation or reduction leads to an increase in the rate of $O_{2}^{•-}$ production. As Eq. (3) shows, this ROS production is proportional to the difference of the redox state (R) from its optimal value (Ropt).

(3) ROS~∣R-Ropt∣.

In addition, the dual agent experiments demonstrated the shift in the redox state towards oxid ation by PCP, and the reversal of the $O_{2}^{•-}$by KCN suggests that the redox optimized balance could be modulated by targeting specific ETC complexes. In this scenario, the redox state (R) of the uncoupled ETC becomes more reduced after adding KCN and moves toward optimal redox state and attenuates ROS production. This is a conundrum! The relationship between ROS and redox state provided in Eq. (3) attempts to resolve this conundrum. That is, even though KCN potentially increases ROS production, paradoxically, in the presence of PCP, it decreases the uncoupling effect on increased ROS production.

Since mitochondria are the major source of $O_{2}^{•-}$ production during the uncoupling of respiration with PCP, we also examined whether the initial surge in $O_{2}^{•-}$ production was amenable to the mitochondria-targeted ROS scavenger, Mito-Tempol. This agent is hitched to the cationic agent TPP+, which pulls the scavenger into mitochondria where it acts as an effective SOD mimetic. The dynamic of mitochondrial $O_{2}^{•-}$ scavenging in the presence of MitoTempol (the growing space between the green and red profiles in Fig. 6) confirms that the initial surge in mitochondrial $O_{2}^{•-}$ production following uncoupling of respiration with PCP is primarily mediated by deranged electron transfer in the ETC. This is also consistent with the observation that complex IV might be the source of electron leak that contributes to the surge in $O_{2}^{•-}$ generation in the initial phase, i.e., when production exceeds the scavenging potential of MnSOD. We further verified that the primary source of the $O_{2}^{•-}$ anion during simulated metabolic stress is mitochondria, because the NOX inhibitor, apocynin, did not alter the cellular ROS production (data not shown).

In conclusion, we used a novel approach that enables us to, for the first time to the best of our knowledge, partition and quantify the dynamics of mitochondrial $O_{2}^{•-}$ production from ETC complexes under different simulated metabolic stress conditions in intact live PAECs. Nanomolar Mito-SOX, to minimize its toxic effects, was used to monitor the rate of mitochondrial $O_{2}^{•-}$ production in the PAECs, which was attenuated by the mitochondria-specific ROS scavenger (Mito-Tempol). We believe this approach could have far-reaching implications for assessment of ROS in physiology and pathophysiology. Therefore, accurate detection of mitochondrial ROS would allow us to establish a diagnostic tool for assessing the role of mitochondrial oxidative stress in the pathogenesis of diseases.

## 5. Limitations

We relied on the concept that MitoSOX is oxidized by $O_{2}^{•-}$ and the oxidation product becomes highly fluorescent upon subsequent binding to mitochondrial DNA over time.^21^ It is possible that as a cationic molecule, MitoSOX uptake into mitochondria can also contribute to direct ROS production by depolarizing ΔΨm. However, the ROS generated in this case was miniscule when compared to the ROS produced from modulating ETC complexes. While nanomolar MitoSOX (used in this study) had no adverse effects,^42^ we recommend a lower nanomolar concentration (200 nM) of MitoSOX to study $O_{2}^{•-}$ production for longer durations.

It should also be noted that MitoSOX uptake increases 10 fold for every 60mV increase in ΔΨm.^21,77^ Since ΔΨm decreases in the presence of uncoupler^78,79^ and ETC inhibitors,^78,80^ the decrease in ΔΨm could impede the uptake of MitoSOX. Therefore, the fluorescence intensities presented could be an underestimate for the levels of the ROS in the inhibited and uncoupled ETC.

Interaction of $O_{2}^{•-}$ and MitoSOX is also affected by the pharmacokinetics of MitoSOX and its binding properties. The initial phase of the intensity profile shows a greatly enhanced fluorescence due to binding of the oxidized MitoSOX to mitochondrial DNA. In the steady state phase of the fluorescence recording, the steady-state signal profile can be possibly due to binding of oxidized MitoSOX to nuclei, which results in apparent nuclear and nucleolar localization (Fig. 2(a)). Due to nuclei binding of MitoSOX and thus, the subsequent diminished mitochondrial uptake of the fluorescent probe, the fluorescence intensity of the mitochondrial compartment becomes saturated and after a while reaches the final level.
